# Interval and Continuous Exercise Training Produce Similar Increases in Skeletal Muscle and Left Ventricle Microvascular Density in Rats

**DOI:** 10.1155/2013/752817

**Published:** 2013-11-26

**Authors:** Flávio Pereira, Roger de Moraes, Eduardo Tibiriçá, Antonio C. L. Nóbrega

**Affiliations:** ^1^Laboratory of Cardiovascular Investigation, Oswaldo Cruz Institute, FIOCRUZ, 21040 900 Rio de Janeiro, RJ, Brazil; ^2^Department of Physiology and Pharmacology, Fluminense Federal University, 24210 130 Niterói, RJ, Brazil; ^3^Laboratory of Exercise Sciences, Biomedical Institute, Fluminense Federal University, Rua Professor Hernani Pires de Mello 1001, Sala 106, 24210 130 Niterói, RJ, Brazil

## Abstract

Interval training (IT), consisting of alternated periods of high and low intensity exercise, has been proposed as a strategy to induce more marked biological adaptations than continuous exercise training (CT). The purpose of this study was to assess the effects of IT and CT with equivalent total energy expenditure on capillary skeletal and cardiac muscles in rats. Wistar rats ran on a treadmill for 30 min per day with no slope (0%), 4 times/week for 13 weeks. CT has constant load of 70% max; IT has cycles of 90% max for 1 min followed by 1 min at 50% max. CT and IT increased endurance and muscle oxidative capacity and attenuated body weight gain to a similar extent (*P* > 0.05). In addition, CT and IT similarly increased functional capillary density of skeletal muscle (CT: 30.6 ± 11.7%; IT: 28.7 ± 11.9%) and the capillary-to-fiber ratio in skeletal muscle (CT: 28.7 ± 14.4%; IT: 40.1 ± 17.2%) and in the left ventricle (CT: 57.3 ± 53.1%; IT: 54.3 ± 40.5%). In conclusion, at equivalent total work volumes, interval exercise training induced similar functional and structural alterations in the microcirculation of skeletal muscle and myocardium in healthy rats compared to continuous exercise training.

## 1. Introduction

Regular practice of moderate aerobic exercise is widely recognized to reduce cardiovascular risk [1]. Since acute exercise is the most important stimulus for increasing myocardial demand, it is not surprising that major structural and functional adaptations occur in the heart consequent to chronic regular exercise training. These adaptations include higher efficiency in myocardial substrate utilization and energetic [2] and enhanced antioxidative stress capacity [3], both potentially involved with the protection against ischemia/reperfusion myocardial damage [3] and infarction [4] developed after exercise training.

Along with metabolic and biochemical myocardial adaptations, major vascular growth and remodeling occur in coronary circulation after exercise training. Enhanced angiogenesis, that is, capillary growth, and vascularization along with altered regulation of coronary blood flow are well-known features of physiological adaptations to chronic exercise and have been extensively revised [5–7]. The balance between metabolic demand and oxygen delivery is maintained along the weeks of training by a dynamic and stepwise combination of early angiogenesis followed by an increase in small and larger arterioles, in part as a consequence of capillaries developing into small arterioles [8].

The increased capillary network and coronary blood flow play an important role in improving aerobic capacity and facilitating oxygen transport, conductance, and muscle extraction, therefore contributing to increase of maximal oxygen uptake and physical performance [3, 9, 10]. Capillary growth in skeletal and cardiac muscle is a complex process mediated by several metabolic alterations occurring during muscle activity and factors linked to increased shear stress induced by blood flow and passive stretch of the vascular smooth muscle tissue [5, 11, 12].

Increasing the intensity of exercise, blood flow also increases [2, 13]. Thus it would be expected that exercise at higher intensities provides a greater stimulus for capillary growth than that of moderate exercise. Surprisingly, the chronic effects of intense or extraneous exercise on myocardial vasculature have been sparsely studied [14, 15] and yet no specific impact of exercise intensity can be derived from those, since the total exercise volume was not controlled. In other words, if an exercise training regime is employed leading to an increased total training volume, a resulting greater physiological adaptation could be attributed to the higher total amount of work and not to the specific impact of the *intensity *itself. On the other hand, a series of well-planned and well-conducted studies have matched the total amount of work in order to investigate the influence of exercise *intensity* itself on a variety of different myocardial measures such as substrate utilization, oxygen consumption [2], and ventricular oxidative stress [4] in rats.

Therefore, despite the physiological and clinical relevance of myocardial vasculature and the unknown effects of higher exercise intensity on this variable, no studies have so far provided evidence on the comparisons between the effects of training regimes of different intensities but the same volume. Thus, the aim of the present study was to assess the effects of two different aerobic exercise programs on myocardial capillary density where exercise intensity was different, but total duration and amount of work were carefully matched. To accomplish this purpose, we submitted a group of Wistar rats to interval training (IT), consisting of alternated periods of high and low intensity exercise and compared the adaptations in skeletal muscle and myocardial microvasculature to those observed in rats submitted to a moderate intensity and continuous exercise protocol (CT) and a control sedentary group.

## 2. Methods

### 2.1. Animals

The experiments were performed with 12- to 14-week-old male WKY rats (Wistar Kyoto, Oswaldo Cruz Foundation animal facilities, Brazil). The animals were housed with controlled light (12 : 12 h light-dark cycle) and temperature (22 ± 1°C) with free access to water and standard rat chow until the day of the experiment. All of the procedures were approved by the Oswaldo Cruz Foundation Animal Welfare Committee (protocol no. P 0034-08) and are consistent with the USA National Institutes of Health *Guide for the Care and Use of Laboratory Animals* (revised 1996).

### 2.2. Experimental Protocols

Thirty WKY rats were randomly assigned to an interval training (IT), continuous training (CT), or sedentary group (SED). The exercise performance tests were conducted immediately before experiment initiation, at six weeks after initiation of the experiment, and at the end of the training program. The intensity of the training program was adjusted after six weeks of training. Twenty-four hours after the last session of exercise training, the rats were anesthetized for intravital microscopy procedures; afterwards, the animals were deeply anesthetized and sacrificed, and the heart and gracilis muscle were immediately removed for subsequent analysis.

### 2.3. Training Program

The exercise training was performed on a low-speed motorized treadmill (Universidade de São Carlos, São Paulo, Brazil) and consisted of a 13-week period of running 30 min per day at no incline (0%), 4 times a week. The training program was preceded by a 10-day adaptation period to the aerobic exercise, during which the running time and speed of the treadmill were gradually increased from 10 min at 15 m/min to the training schedule described below. The exercise performance tests consisted of graded exercise on the treadmill at no incline, starting at 10 m/min with increments of 1 m/min every 1 min up to the maximal running speed attained by each rat (exhaustion). In the CT group, the treadmill speed was incrementally increased to attain 70% of maximal exercise capacity. In the IT group, the animals ran 1 min intervals at 90% of maximal exercise capacity, followed by 1 min intervals at 50% of maximal exercise capacity [16]. Therefore, the total volume of exercise (intensity × duration × frequency) was matched among protocols since the mean intensity during IT was 70% max, the same intensity applied throughout the CT. In order to assure that total energy expenditure was indeed matched during IT and CT, we measured total oxygen consumption (metabolic treadmill-AVS, São Paulo, Brazil) during one typical session of IT (*n* = 5) and one typical session of CT (*n* = 5), both lasting 30 min.

The sedentary rats walked freely on a nonmoving treadmill 30 min per day 4 times a week during the 13-week period.

### 2.4. Intravital Fluorescence Video Microscopy

The animals were anesthetized with pentobarbital (75 mg/kg, i.p.), intubated, and artificially ventilated with ambient air using a small animal ventilator (Ugo Basile model 7025, Varese, Italy). The right jugular vein was catheterized to permit injection of the anesthetic agents and fluorescent dye. The central temperature was monitored with a rectal probe, and the body temperature was kept at 38 ± 0.5°C with a homeothermic blanket system (Harvard Apparatus, Boston, USA).

The ear skin was scraped, and the animals were placed on their back on a Plexiglas pad. The gracilis muscle was exposed through an incision in the right thigh and was covered with an oxygen-impermeable plastic wrap. The animals were then placed under an upright, fixed-stage intravital microscope (Olympus BX51, WI, USA) coupled to a CCD digital video camera system (Optronics, Goleta, USA). A 10x objective magnification was used, resulting in a total magnification of 100x at the video monitor. After intravenous injection of 0.15 mL of 5% fluorescein-isothiocyanate- (FITC-) labeled dextran (molecular weight 150,000), microscopic images of the muscle and the skin were successively collected, and the capillaries were quantified with Saisam software (Microvision, Evry, France). The functional capillary density, which was defined as the total number of spontaneously perfusedcapillaries per square millimeter of surface area (1 mm^2^), was determined from several random microscopic fields over a period of 4 minutes.

### 2.5. Histochemical Analysis

#### 2.5.1. Gracilis Muscle

The tissue samples were dehydrated in a graded series of ethanol (70%, 95%, and 100%) and embedded in paraffin. The paraffin blocks were cut into 5 *µ*m sections and stained with FITC-conjugated *Griffonia simplicifolia* I lectin at a 1 : 150 dilution in a dark humidified chamber at room temperature for 30 min to visualize the capillary endothelial cells. The structural capillary density (number of capillaries per mm^2^) and structural fiber density (number of muscle fibers per mm^2^) were assessed with a confocal microscope (Olympus BX51 and FluoView SV 300 scanning unit, Olympus, USA) and analyzed with Saisam software. The structural capillary density of the skeletal muscle was evaluated with at least 12 microscopic fields randomly selected from the tissue sections. The capillary-to-fiber ratio, which is considered to be an anatomic index of angiogenesis, was calculated by dividing the capillary density by the fiber density.

#### 2.5.2. Left Ventricle

The tissue samples were dehydrated in a graded series of ethanol (70%, 95%, and 100%) and embedded in paraffin. The left ventricular structural capillary density was determined according to the orientator method as previously described [17]. Briefly, the orientator method is an approach for generating isotropic, uniform, and random sections of biological specimens that allows for the quantitative study of three-dimensional anisotropic structures from the two-dimensional sections. Although the myocardium is an anisotropic structure, isotropic sections are required for a stereological study. The technique was performed by cutting the organ according to the “orthrip” method [18], which turns the sample into uniformly isotropic sections by dividing the fragment three times consecutively; the first section is random, the second section is orthogonal to the first, and the third section is orthogonal to the second. The paraffin blocks were cut in 5 *µ*m sections and stained with FITC-conjugated *Griffonia simplicifolia* I lectin at a 1 : 150 dilution in a dark humidified chamber at room temperature for 30 min. For each animal, at least 7 microscopic fields were randomly examined from 3 different tissue sections. The volume density of the capillaries (*V*_*v*[cap]_) was calculated as follows: *V*_*v*[cap]_ = *P*_*p*_/*P*_*T*_ (%), where *P*_*p*_ is the number of points that are coincident with a capillary and *P*_*T*_ is the total number of test points (*P*_*T*_ = 56 in the present case). The fiber volume density (*V*_*v*[fib]_) was similarly calculated. The ratio of the capillary volume density to the fiber volume density (*V*_*v*[cap]_/*V*_*v*[fib]_) was calculated to negate any influences of cardiac hypertrophy on myocardial capillary density.

### 2.6. Citrate Synthase Activity

Tissue samples from the gracilis muscle were obtained to biochemically analyze citrate synthase activity to determine the effectiveness of the exercise training. The muscle samples were immediately frozen in liquid nitrogen and stored at −80°C until processing. Citrate synthase activity was measured from whole muscle homogenate using the spectrophotometric method published by Srere [19].

#### 2.6.1. Drugs

The following drugs were used: sodium pentobarbital, pancuronium bromide, FITC-labeled dextran, and FITC-conjugated *Griffonia simplicifolia* I lectin (Sigma Chemical Co., St. Louis, USA).

#### 2.6.2. Statistical Analysis

The results are expressed as the mean ± SD for each group, and comparisons between the different groups were made with one-way analysis of variance (ANOVA). If a significant difference was detected by ANOVA, the Bonferroni test was used to identify the statistically significant differences. Differences with a *P* value less than 0.05 were considered significant. All calculations were made by computer-assisted analyses with a commercially available statistical package (GraphPad Instat 5.0, GraphPad software).

## 3. Results

### 3.1. Energy Expenditure during IT and CT

Total running distance (IT: 496 ± 37 m; CT: 499 ± 60 m; *P* > 0.05) and total oxygen consumption during the exercise session (IT: 1,075 ± 184 mL; CT: 1,036 ± 299 mL; *P* > 0.05) were similar between training protocols.

### 3.2. Exercise Training Efficacy, Body Weight, and Citrate Synthase Activity

**Figure 1:**
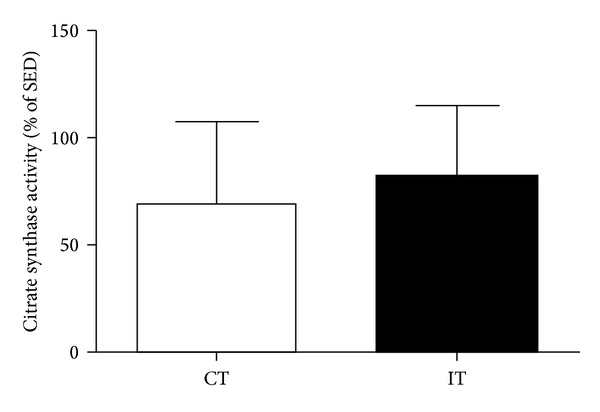
Percentage increase in citrate synthase activity in the gracilis muscle of continuous-trained (CT) and interval-trained (IT) rats, when compared to sedentary (SED) rats. The values are the mean ± SD of 10 experiments.

After six and 13 weeks of exercise, both exercise methods (CT and IT) induced several changes indicative of a trained state in rats (Table 1 and Figure 1). The maximal velocity achieved in the treadmill endurance test was significantly higher (*P* < 0.05) in the trained animals than in the SED animals (Table 1); the observed increase in velocity was similar for the CT and IT groups at both six and 13 weeks of training. Additionally, body weight was lower in the CT and IT groups than in the SED group at the end of the training period. Moreover, gracilis muscle homogenate citrate synthase activity was significantly higher in both the CT and IT rats (321 ± 69 and 342 ± 61 nmol/mg/min, resp.; *P* < 0.001) than in the SED animals (189 ± 100 nmol/mg/min). The percentage increase in citrate synthase activity was of 69.0 ± 38.3 and 81.2 ± 32.9 for the CT and IT groups, respectively (Figure 1). These results indicate that the exercise programs enhanced the skeletal muscle oxidative capacity of the exercise groups relative to the control group.

### 3.3. Functional Capillary Density in Skeletal Muscle and Skin

**Figure 2:**
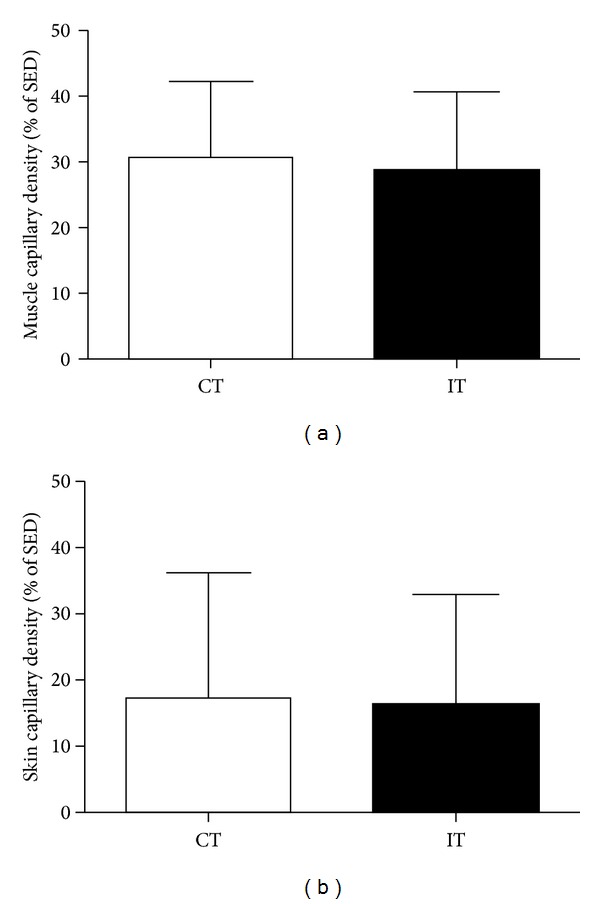
Percentage increase in functional capillary density in the gracilis muscle (a) and skin (b) of continuous-trained (CT) and interval-trained (IT) rats, when compared to sedentary (SED) rats. The values are the mean ± SD of 10 experiments.

Both the CT and IT groups had a significant increase in the functional capillary density of their skeletal muscle (282 ± 7 and 283 ± 8 capillaries/mm^2^, resp.; *P* < 0.001), compared with that of the sedentary animals (SED: 216 ± 7 capillaries/mm^2^). The percentage increase in muscle functional capillary density was of 30.6 ± 11.7 and 28.7 ± 11.9 for the CT and IT groups, respectively (Figure 2). There was no significant difference in the functional capillary density of the skin for any of the groups. The percentage increase in skin functional capillary density was of 17.3 ± 19.1 and 16.3 ± 16.9 for the CT and IT groups, respectively (Figure 2).

### 3.4. Structural Capillary Density in Skeletal Muscle and the Left Ventricle

**Figure 3:**
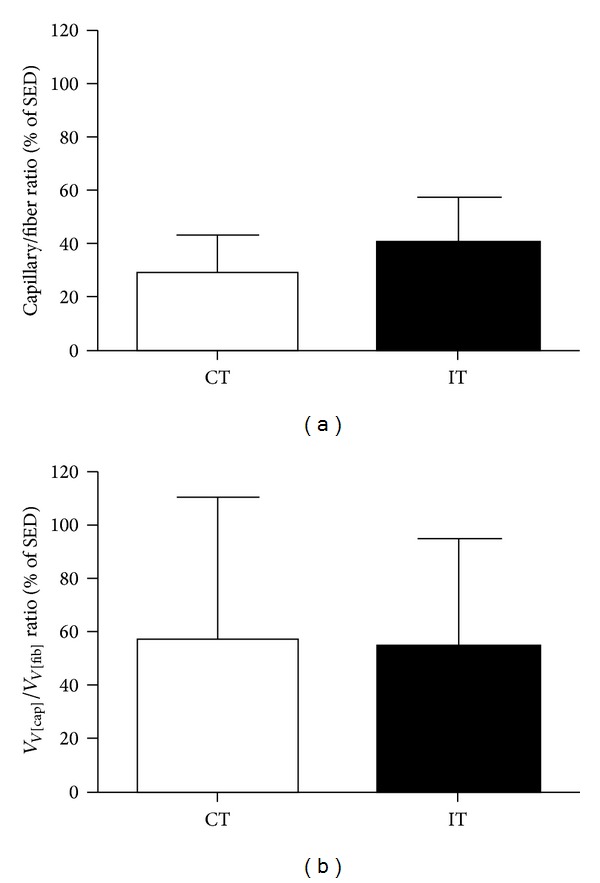
Percentage increase in capillary-to-fiber ratio in the gracilis muscle (a) and capillary volume density-to-fiber volume density ratio in the left ventricle (b) of continuous-trained (CT) and interval-trained (IT) rats, when compared to sedentary (SED) rats. The values are the mean ± SD of 10 experiments.

Compared to the control group, both exercise training regimens similarly increased the capillary-to-fiber ratio in skeletal muscle (CT: 1.66 ± 0.06 and IT: 1.80 ± 0.09 versus SED: 1.28 ± 0.06; *P* < 0.001). The percentage increase in muscle structural capillary density was of 28.7 ± 14.4 and 40.1 ± 17.2 for the CT and IT groups, respectively (Figure 3). For the left ventricle, the structural capillary density was evaluated in sections that were obtained with the “orientator” method [17], which allow for the visualization of unbiased and uniformly isotropic structures in anisotropic tissues, such as the heart. The capillary volume density-to-fiber volume density ratio (*Vv*_[cap]_/*Vv*_[fib]_) in the left ventricle of both exercise groups was increased to a similar magnitude, when compared to that in the control group (CT: 0.60 ± 0.19 and IT: 0.56 ± 0.11 versus SED: 0.41 ± 0.14; *P* < 0.05). The percentage increase in left ventricle structural capillary density was of 57.3 ± 53.1 and 54.3 ± 40.5 for the CT and IT groups, respectively (Figure 3).

## 4. Discussion

The most important novel findings of the present study are as follows: (1) CT and IT increased exercise endurance and muscle oxidative capacity and attenuated body weight gain to the same extent; (2) CT and IT increased similarly functional and structural alterations in the microcirculation of locomotor skeletal muscle and of the myocardium of rats. These results suggest that IT mode is equivalent to CT in increasing exercise capacity and in inducing microvascular adaptations in both skeletal and cardiac muscles, when total training volume is matched supporting the concept that total energy expenditure, and not exercise intensity per se, is the major physiological stimulus, at least for chronic changes in skeletal and myocardium microcirculation.

The large increase in oxidative enzyme citrate synthase, performance, and capillary density produced by both IT and CT in the present study is consistent with numerous studies of endurance trained athletes and animals [2, 16, 20, 21]. Many cross-sectional studies have demonstrated that trained endurance athletes present skeletal muscle oxidative enzyme activities that are much greater than those of their sedentary counterparts [10, 12, 22]. These increases in oxidative capacity are associated with increases in skeletal muscle mitochondrial content via mitochondrial biogenesis [23] and are consistent with the previously shown relationship between capillarity and mitochondrial content [9, 24, 25].

Previous studies investigating the influence of different exercise intensities on improvements of microcirculation and *V*_O_2peak__ have met criticism because of the insufficient equation between intensity and duration [26–28]. Thus, consensus about the most efficient exercise intensity to increase microcirculation in cardiac and skeletal muscle is still lacking. The hypothesis was that interval exercise including periods of high intensity exercise would promote similar increases in skeletal and myocardial muscles when compared to continuous training, as long as total training volume—energy expenditure—is kept equivalent.

Previous studies combined provide evidence that the adaptations to exercise training may vary depending on training protocols, exercise intensity, and total training volume regarding the effect on microcirculation and oxidative capacity [28–32]. The results from these studies are equivocal, reporting similar increases in the maximal activities of mitochondrial enzymes and microcirculation after interval and continuous training only [27, 29, 33, 34]. To our knowledge, the present study is the first to directly compare changes in oxidative capacity and microcirculation in skeletal and cardiac muscle with matched-work training protocols that lasted several weeks.

Despite the established concept that exercise training adaptations are primarily dependent on training volume, at least two previous studies have reported greater improvements in aerobic fitness in healthy humans after interval training compared with continuous training, regardless of total work being matched [35, 36]. Neither one investigated the effects on skeletal muscle or myocardial microvasculature. Nevertheless, differences in the training intensity, duration, and subject population used may explain these contrasting results. For example, in the study by Gorostiaga et al. [36], while the interval training group cycled at 100% *V*_O_2peak__, the continuous training group cycled at only 50% of *V*_O_2peak__, a much lower training intensity. This may have contributed to their findings of greater improvements in *V*_O_2peak__ after high intensity interval training than in moderate intensity continuous training.

The magnitude of improvement in performance after interval and continuous training programs may also depend on other training variables, such as the length of the training regimen and prior fitness status of the subjects. The study by Eversten et al. [35] reported greater improvements in cross-country skiers submitted to high intensity exercise as compared to a moderate intensity protocol at similar training volume. However, the subjects were well-trained endurance athletes (*V*_O_2peak__: 73 ± 4 and 58 ± 2 mL/kg/min for the males and females, resp.) and trained over a 5-month off-season period, thus encompassing a much larger total training volume than the present study.

Other earlier reports [26, 28, 34, 37, 37–40] have concluded that high intensity exercise training is superior to moderate intensity with regard to increasing aerobic capacity, running speed, and several biochemical and metabolic variables. However, most of them have not matched total exercise volume—energy expenditure—between protocols, precluding any direct conclusion regarding the specific role of intensity in these physiological adaptations to exercise training. In the few studies where total training volume was indeed controlled, different animal models were used, making a direct comparison with our studies quite difficult. For example, Haram et al. [37] studied an artificial selective breeding of rats that developed low intrinsic aerobic capacity and a metabolic syndrome phenotype. In others, such as the one by Moreira et al. [20] where total exercise volume was matched, no difference between high intensity and moderate intensity exercise on muscle adaptation, was found.

The present study reveals that in normal rats, IT induces significant changes in the structure and function of the gracilis muscle. These changes are similar to those induced by CT. The structural and functional adaptations within our rats gracilis muscle are consistent with those in earlier reports [21, 37, 41, 42]. For example, the observed values for capillary to fiber ratio of IT and CT are in line with previously published reports [2, 16, 20, 35]. Such changes within the muscles explain, at least in part, the substantially increased capacity for aerobic work observed after training.

The present results revealed a marked increase in the number of spontaneously perfused capillaries in locomotor skeletal muscle in both the CT and IT groups as assessed by intravital video microscopy. In contrast, there was no change in the functional capillary density of the ear skin. Thus, these results suggest that both CT and IT increase perfusion only in tissues that are actively participating in exercise and are subjected to repeated increases in metabolic demand, blood flow, and vascular shear stress.

The mechanisms underlying exercise-induced angiogenesis and vasculogenesis in skeletal muscle are well defined [43, 44]. By contrast, angiogenesis in the adult heart is a controversial issue. In general, studies have reported an increase in myocardial capillary density in young animals subjected to endurance training but no change or a decrease in adult animals, even though the training consisted of weeks or months [45]. In a comprehensive study that evaluated the time course of capillary growth during training, the authors reported that cardiac vascular synthesis and remodeling occurred in young animals subjected to endurance training within the first 3 weeks of exercise [8]; thus capillary growth may be an early and transient feature of training that may have been missed by other long-term studies with a single end point. In the present study, even after a long period of either CT or IT, there is still a significant increase in the structural capillary density in the myocardium of exercised rats, compared to that in the sedentary control animals.

Finally, it is worth mentioning that the vascular adaptations, as well as their underlying mechanisms, induced by exercise training, are not consistently distributed along the arterial tree [46]. Moreover, the improvement of vascular endothelial function induced by exercise training is not limited to the active muscle vascular beds such as the skeletal, respiratory, and cardiac muscles [47]. Actually, these adaptations in the vasculature of nonworking skeletal muscle, brain, viscera, and skin can also differ substantially [48]. Thus, the results of our study are limited to active muscle vascular beds including the skeletal and cardiac muscles.

In conclusion, we found that when total exercise volume is matched, interval training that included periods of high intensity exercise produces similar adaptations to moderate and constant exercise with regard to effects on exercise capacity, skeletal muscle citrate synthase effects, and microcirculatory beds of skeletal muscle and myocardium.

## Figures and Tables

**Table 1:** Exercise performance test results and body weight of sedentary (SED), continuous-trained (CT), and interval-trained (IT) rats.

	Velocity (m/min)			Body weight (g)		
	0 weeks	6 weeks	13 weeks	0 weeks	6 weeks	13 weeks
SED	24.6 ± 1.3	19.4 ± 1.1	14.6 ± 1.8	276 ± 5	371 ± 10	436 ± 12
CT	24.8 ± 1.5	28.0 ± 1.0*	32.3 ± 1.3*	270 ± 6	356 ± 9	396 ± 10*
IT	24.7 ± 1.4	28.8 ± 3.3*	32.8 ± 1.3*	272 ± 5	350 ± 5	381 ± 8*

The values are the mean ± SEM; *n* = 10 for all groups.

*P < 0.05 compared to the sedentary rats (SED).
